# Is Tuberculosis a Disease of Environment Only?

**Published:** 1904-11

**Authors:** H. McHatton

**Affiliations:** Macon, Ga.


### IS TUBERCULOSIS A DISEASE OF ENVIRONMENT ONLY?*

BY H. MCHATTON, M.D.,

MACON, GA.

In taking up the question of the prevention ot tuberculosis, it would be well to review briefly the changes in opinion that have taken place in our profession in the past thirty years.

Thirty years ago, tuberculosis was considered inherited and incurable. Then gradually the possibility of a tuberculosis following a bronchitis or pneumonia where there was no inherited taint, then the possibility of a contagion, the probability of a contagion, the absolute proof of contagion. The question of heredity, inherited predisposition making one more prone to the disease, and a gradual shading of these opinions as science advanced, until at present in my opinion, the advanced students in this line consider this disease a specific contagious disease, in which direct heredity bears a most infinitesimal part, if any—a disease spread by taking into the body in various ways the specific germs; consequently, by proper environment, absolutely preventable, and in a vast majority of cases curable in the early stages.

In support of the above opinion, allow me to make use of some facts gathered for a paper on this subject read before the Congress of Tuberculosis ot 1902, in which I propose to show that under certain types of environment, large numbers of people of various races were rendered tubercular or non-tubercular, and this by the observance or non-observance of the ordinary rules of hygiene.

Prior to 1860, a vast majority of the negroes in the Southern States and in Cuba lived on plantations. These plantations were to all intents and purposes isolated communities of from one hundred to five hundred inhabitants. The negro quarters were invariably placed in the healthiest available spot, the houses usually consisted of two rooms and were never overcrowded; each room had its open fireplace and fuel was abundant—thus the best ventilation was secured all the year around. There was sample air-space be-

*Address before Georgia State Commission on Tuberculosis.

tween the houses and the quarters always had an abundance of shade-trees. On account of the climate, most of the idle time was spent out of doors. Absolute cleanliness was enforced in and around the quarters. Each house, whether wood or brick, had its coat of whitewash inside and out three or four times a year, and oftener if any disease threatened. Communication between plantations was not encouraged, and when any disease became prevalent, absolutely prohibited. The water-supply was always a source of solicitude, and the best obtainable secured. All innocent amusements, so dear to the heart of the African, were not only permitted but encouraged. Dissipation of all types was prohibited, and under existing conditions this prohibition could be easily enforced. There was no mental solicitude, no competition in the ordinary sense and no care for the future. Work was never excessive, always in the best hygienic surroundings and of the healthiest type, also in proportion to the age, strength and sex of the individual. Food was cooked in the plantation kitchen, abundant, nutritious and well prepared. Clothing was always sufficient and in accordance with the season. As soon as a woman was over her lying-in period the infant was transferred to the nursery, where it was only nursed at regular intervals, and fed according to its age. Each negro reported in the morning, sick or well; if sick, he went to the infirmary. The doctor visited each plantation once or twice a week, and was called in any emergency. Thus each negro who was not physically perfect was under constant medical supervision.

In Cuba the arrangement of the quarters was different, they being on the type of the old Spanish barracoon. All other surroundings were practically the same as in the South. On all plantations early to bed and early to rise was the rule, with a long midday rest and meals by the clock. Among the very small percentage of negroes who lived in the cities the plantation rules were carried out as far as practicable. I can state without fear of contradiction that no race of human beings ever lived as healthy a life as the plantation negro in the South. Tuberculosis was practically unknown. I was born on a plantation in Louisiana, and lived on plantations in the South and in Cuba until I was twenty-three years old, and never saw a tubercular negro.

Freedom came. The entire proposition is reversed. They flock to the cities with about as much capacity for self-care as children; hard work, inadequate clothing, poor food, poor houses, overcrowding in insalubrious localities, no attention to the most elementary laws of hygiene, all the mental cares and solicitude that freedom brings. Scant wages, spent in the lowest and most degrading vices at the sacrifice of absolute necessities, irregular hours, with practically no medical attention, or care in sickness. Today the town negro in the South is probably the most tubercular being in the United States.

It is nothing unusual in my practice to see entire families wiped out. In 1900, twenty-one per cent. of the total negro mortality in Macon was from tuberculosis.

About 1860 Spain entered into a coolie contract with China. Under this contract about two hundred and fifty thousand Chinese were imported into Cuba in the '60s and '70s, the vast majority going to the plantations where they were put under the identical surroundings of the negro.

These coolies were all men ranging from eighteen to thirty years of age. They were the scum and offscourings of the cities—principally Canton and Macao—bringing with them the city diseases as well as the city vices, for which China is noted. For obvious reasons, both vices and diseases could be rapidly controlled, and these men were soon put in nearly as good a physical condition as the negro. There was always, even with the most careful selection, a fair percentage of tubercular cases in each gang of coolies. I can safely say that within the first year this was eliminated either by death or cure. After that there was no more development of the disease. The half-breeds from the mixture of the two races, of which there was a large number, were equally immune.

About 1790, there landed at Trujaillo, on the Carribean Sea, a party of Spanish emigrants. This party consisted of members of ten families of the Spanish nobility—families who were so tuberculous that they decided to emigrate rather than become extinct.

They worked their way in the course of time across Central America and settled on the Pacific slope not far from Tegucigalpa, and at an altitude of about twenty-five hundred feet in probably one of the most even and healthy climates in the world. They have always been purely agricultural and pastoral. Even today there is not a road leading to this colony—nothing but trails, and it is a journey of days to reach them from the nearest port. Their village is built in accordance with the climatic requirements. They hold themselves far above the surrounding Indians, and there has been practically no intermarriage between them and their neighbors. They present the purest strain of Spanish blood in America.

The Indians ten or fifteen days' ride from this colony never fail to speak of it always as "El pueblo de los blancos," "the village of the whites," and to extol the physique and endurance of the men as well as the beauty and virtue of the women, which opinion the few specimens that I saw fully upheld.

The history of these people was given me in a personal interview by Don Torencio Sierra, President of Honduras, and a most highly educated gentleman.

Dr. O. B. Hunter, of San Pedro Sula, a graduate of Tulane University, learning their history, became so much interested in them that he spent sometime in their village with the sole object of learning their present condition. He met some of the children of the original immigrants, now old men and women, who in every way corroborated the above history.

Dr. Hunter informs me that they are the finest race of people in Central America. After careful inquiries he could get no history of tuberculosis for a long period back, and at present none of them give physical evidence of this disease in any of its numerous forms.

The use of the illustrations in the case of the Chinese and Spaniard is simply to show that the history of our Southern negro is not unique. In him we have one of the most remarkable instances in the history of the world in regard to tuberculosis, one of which there is no question, one with which each of the oldest members of this commission is familiar, one in which the question of heredity can not enter, one in which from an absolutely nontubercular race, the negro has been changed in forty years into one of the most tubercular races in the world.

If the planter in the South could keep his negroes in the condition that he did, what should be expected of the result of the labors of this commission in the course of time—backed by the State of Georgia and seconded by every physician in our land.

Similar commissions have already reduced the mortality of tnberculosis very markedly in other countries.

The main thing that you will have to combat is an apathy of the people when it comes to a question of hygiene.

They will have to be educated up to the fact that consumption is acquired almost invariably from the sputa of the consumptive; that there is no contagion in the ordinary sense of the word from his person; that the disease is not inherited; that in the early stages, it is usually curable, and that sunshine and fresh air are the greatest natural enemies of the tubercle bacillus.

